# Assessment of the Psychological Situation in Adults with Congenital Heart Disease

**DOI:** 10.3390/jcm9030779

**Published:** 2020-03-13

**Authors:** Caroline Andonian, Jürgen Beckmann, Peter Ewert, Sebastian Freilinger, Harald Kaemmerer, Renate Oberhoffer-Fritz, Martin Sack, Rhoia Neidenbach

**Affiliations:** 1 Department of Pediatric Cardiology and Congenital Heart Disease, German Heart Center Munich, Technical University Munich, 80636 Munich, Germany; ewert@dhm.mhn.de (P.E.); freilinger@dhm.mhn.de (S.F.); Kaemmerer@dhm.mhn.de (H.K.); oberhoffer@dhm.mhn.de (R.O.-F.); neidenbach@dhm.mhn.de (R.N.); 2 Department of Sport and Health Sciences, Chair of Sport Psychology, Technical University Munich, 80809 Munich, Germany; juergen.beckmann@tum.de; 3 School of Human Movement and Nutrition Sciences, University of Queensland, Brisbane, Queensland 4067, Australia; 4 Clinic and Policlinic for Psychosomatic Medicine and Psychotherapy, School of Medicine, Technical University Munich, 81675 Munich, Germany; m.sack@tum.de

**Keywords:** adults with congenital heart disease, psychological situation, illness identity, prevention, anxiety, depression

## Abstract

Background: Due to advances in the diagnosis and treatment of congenital heart disease (CHD), the number of adults who are surviving with congenital heart disease (ACHD) is constantly growing. Until recently, the psychological effects of CHD had been widely neglected. Current research provides evidence for an increased risk of emotional distress in ACHD. The concept of illness identity attempts to explain how patients experience and integrate their CHD into their identities. The present study investigated illness identity in relation to clinical parameters and psychological functioning. Psychometric properties of the German version of the Illness Identity Questionnaire (IIQD) were examined. Methods: Self-reported measures on illness identity and psychological functioning (HADS-D) were assessed in a representative sample of 229 ACHD (38 ± 12.5 (18−73) years; 45% female) at the German Heart Center Munich. Descriptive analyses and multiple regression models were conducted. Confirmatory factor analysis was performed to validate the IIQD. Results: The IIQD demonstrated good reliability. The originally-postulated four-factor structure could not be replicated. Anatomic disease complexity and functional status significantly influenced illness identity. Illness identity accounted for unique variances in depression and anxiety: Maladaptive illness identity states (i.e., , engulfment and rejection) were associated with higher emotional distress, whereas adaptive illness (i.e., , acceptance and enrichment) identity states were linked to lower emotional distress. Conclusions: Illness Identity emerged as a predictor of emotional distress in ACHD. Findings raise the possibility that interventions designed to target a patient’s illness identity may improve psychological well-being and cardiac outcomes in ACHD.

## 1. Introduction 

Due to advances in medical care and treatment of congenital heart defects (from CHD) over the last decades. the population of adults with relatively normal life expectancy is continuously growing. Worldwide, 1.35–1.5 million children are born with CHD each year. and more than 90% of them reach adulthood [1].

Yet, the majority of adults with congenital heart disease (ACHD) is chronically ill due to lifelong residua and sequelae of the underlying heart disease [2,3,4]. However, medical restraints are not the adults’ only source of suffering, a sequence of life-altering experiences, such as early trauma, numerous hospitalizations and continuous medical emergencies may seriously impact their mental health status. 

Current empirical evidence suggests that ACHD involves a significantly higher risk for displaying emotional distress, such as depression and anxiety [5]. Emotional distress is known to compromise quality of life (QOL), along with impeded psychosocial functioning and decreased life satisfaction in ACHD [6]. Moreover, research performed in the field of acquired heart disease suggests that chronic emotional distress negatively influences cardiovascular health outcomes, and can even lead to premature mortality [7,8,9,10].

**Figure 1:**
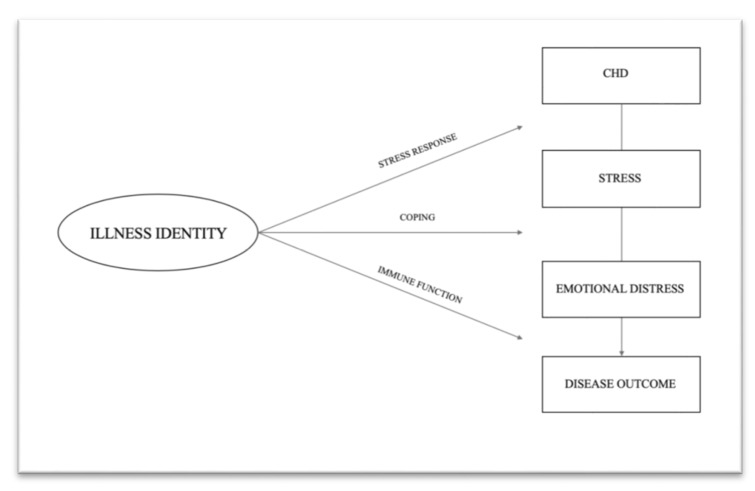
Hypothetical Pathways by Which Illness Identity Influences Emotional Distress and Disease Outcome; Derived and Modified According to Carver and Vargas [12].

To date, differences in psychological functioning could not be sufficiently explained by the underlying severity of disease in ACHD [11]. Apparently, some patients succeed in overcoming disease-related challenges, while others experience substantial difficulties that lead to increased emotional distress. In an attempt to predict chronic disease management, Oris et al. [11] recently introduced the concept of illness identity, which stresses the importance of integrating the illness into one’s sense of self in order to enhance psychological adjustment and physiological health outcomes in ACHD. Figure 1 depicts the hypothetical pathways among CHD, illness identity, psychological functioning and cardiovascular health. In this figure, illness identity functions as a moderator variable, potentially influencing the amount of stress arising from the complications associated with a CHD (path 1), the degree and severity of emotional distress resulting (path 2), and the way of how emotions are translated into potential physiological responses (path 3) [12].

Oris et al. [11] distinguish among four different states of illness identity, i.e., rejection, engulfment, acceptance and enrichment. The first two forms represent a lack of illness integration, and entail maladaptive responses, such as avoidance, denial or excessive concern. Rejection refers to a state where individuals attempt to evade or escape feelings of distress as they refuse to experience their illness as part of their identities [13]. In contrast to rejection, some patients completely renounce their identities, and become exclusively self-defined by their illness, as they pay exclusive attention to their symptoms and restrictions in daily life. Up to now, scientific research has sparsely addressed the topic of identity loss due to chronic illness. Acceptance and enrichment represent adaptive forms of illness integration [11]. 

Instead of struggling with their illness, patients successfully cope with their illness by learning to accept and accommodate to their impairments in adaptive ways [14]. Individuals who accept their illness may find the right balance between devoting attention to their illness and constructing valued lives besides their illness [14]. It is argued that acceptance enables optimal processes of self-management, in which individuals actively engage to manage their chronic illness [15]. Enrichment refers to a state in which individuals benefit from their struggle with a chronic illness. Positive changes include increased life appreciation, personal strength, more meaningful relationships, or spiritual growth [16]. Oris’ research provided important evidence that illness identity might be an important explanatory variable in predicting health-related outcomes in ACHD, and could potentially offer a useful tool in clinical practice [11]. However, the influence of clinical parameters has not been sufficiently examined, and findings should only cautiously be transferred to the highly heterogeneous population of ACHD. 

The primary aim of the present study was to examine the psychological situation of ACHD by (I) validating the German version of the illness identity questionnaire (IIQD) with a representative sample of ACHD at the German Heart Center Munich, (II) investigating potential differences in illness identity states with regard to sociodemographic and clinical variables, (III) exploring unique relationships between the four illness identity states and emotional distress in terms of anxiety and depression. Based on the findings regarding the addressed explanatory psychological variables, we attempt to outline a comprehensive and holistic approach towards dealing with and treating the growing population of ACHD. 

## 2. Materials and Methods

### 2.1. Sample

The present study was part of the nationwide MERLIN-CHD initiative, which constitutes the first large-scale attempt in German-speaking countries to investigate the health situation of ACHD and its effects on lifestyle behaviors and physical outcomes. It was carried out in a cross-sectional setting. Study participants were enrolled at the German Heart Center Munich, a large-volume university hospital which covers a large spectrum of AHCD ranging from simple to complex severity. Inclusion criteria were: (1) Confirmed diagnosis of CHD, according to the definition of Thiene and Frescura [17]; (2) participants aged 18 years or older; (3) necessary physical and cognitive capabilities to complete self-report measures; (4) German-speaking. Participants were excluded if they did not fulfill age requirements, or had severely impaired cognitive abilities. An a priori power analysis calculated an approximate number of 200 participants, assuming a non-responder rate of 20%, which is common in similar surveys. 

### 2.2. Data Collection Procedures

The recruitment goal was to enroll a total number of 200 ACHD. Questionnaires were distributed in inpatient and outpatient units to maximize response. All patients received a study package consisting of: (1) study information, (2) informed consent and (3) set of questionnaires. Informed consent was obtained from each patient, and procedures were performed in accordance to the Declaration of Helsinki [18]. The study has been approved by the Ethical Committee in June 2019 (158/19 S). In addition, medical records were reviewed for each patient in order to detect eventual variations in psychological variables in relation to medical parameters. Data on primary CHD diagnosis, disease complexity and functional status were extracted and recorded separately. The process of data collection started in June 2019 and ended in July 2019. Ethical Statement: The study was part of the nationwide MERLIN CHD initiative. It was approved by the ethical committee of the Technical University of Munich in June 2019 (56/S6).

### 2.3. Measures

Three domains were measured: (1) sociodemographic and clinical variables, (2) illness identity and (3) emotional distress in terms of depression and anxiety. All outcome variables were assessed by standardized questionnaires. 

#### 2.3.1. Demographic and Clinical Information

Sociodemographic variables were assessed with a questionnaire. Clinical information, including CHD diagnosis, functional status, surgical status and cyanosis, was obtained from medical records. CHD diagnosis was divided into three groups according to the classification of CHD severity by Warnes et al. [19].

#### 2.3.2. Illness Identity

The illness identity questionnaire is a self-report measure which was initially developed and validated by Oris et al. [20] in individuals with type 1 diabetes. A former study performed within the international research project APPROACH-IS could further demonstrate high factorial validity and reliability of the IIQ in ACHD [11]. Based on confirmatory and exploratory factor analysis, four illness identity states were formulated: rejection, engulfment, acceptance and enrichment. Respectively, Cronbach alpha values for ACHD were 0.75 for rejection, 0.83 for acceptance, 0.92 for engulfment and 0.95 for enrichment. All factor correlations are below 0.8 and indicate a high discriminant validity. To achieve high equivalence between the original English questionnaire (IIQ) and the translated German version (IIQD), forward- and back-translation was performed by two independent bilinguals. Participants were asked to indicate how much they agreed or disagreed with each of a total of 25 statements on a five-point Likert scale, ranging from 1, i.e., strongly disagree to 5, i.e., strongly agree. The IIQ includes a five-item rejection scale, seven-item enrichment scale, five-item acceptance scale and eight-item engulfment scale.

#### 2.3.3. Depressive and Anxiety Symptoms

The Hospital Anxiety and Depression Scale (HADS) was used to assess patients’ perceived emotional distress, including depressive and anxiety symptoms [21,22]. The HADS is specifically developed and confirmed for its use in medical populations [23]. Cronbach alphas were 0.83 for depressive symptoms in ACHD and 0.87 for anxiety symptoms. The HADS consists of a fourteen-item scale, which combines two seven-item sub-scores for depression and anxiety, respectively. Each item is scored on a response-scale with four alternatives ranging between 0 and 3. All scores are summed up, with elevated scores indicating more emotional distress. 

### 2.4. Statistical Analysis

The statistical analysis included different methodologies to follow the multiple objectives mentioned above. First, descriptive statistics were calculated measuring univariate coefficients of central tendency and distribution. In order to evaluate the relatively-new scale for Illness Identity, psychometric properties of the underlying scale dimensions were analyzed by assessing reliability and factorial structure. While reliability was measured by means of Cronbach’s Alpha, factorial structure was tested by applying a Confirmatory Factor Analysis to the four-dimensional, twenty-five-item version of the IIQ. Model fit was evaluated using established fit indices, such as root mean square error of approximation (RMSEA), comparative fit index (CFI), as well as the Tucker Lewis Index (TLI). Differences in illness identity states according to clinical parameters were evaluated by means of multivariate analysis of variance (MANOVA). Multivariate analysis was conducted, since Confirmatory Factor Analysis indicated significant correlations between the dependent variables. Additionally, multiple regression analysis was used to predict different manifestations in illness identity by considering sociodemographic, clinical and psychological variables. In order to analyze associations of illness identity and psychological functioning by means of depression and anxiety, intercorrelations and multiple regression analysis were used.

Since no significant correlations between sociodemographics (age, gender) and variables of illness identity or psychological functioning were observed, bivariate Pearson coefficients were calculated. For multiple regression analysis, anxiety and depression were predicted using sociodemographic and clinical variables, as well as illness identity. For all models, multiple regression analysis was modeled by means of ordinary least squares parameter estimation. Statistical analysis was performed using SPSS 25.0 (IBM Inc., Armonk, NY, USA). The dedicated SPSS-module for structural equation analyses and model building AMOS v. 22.0 was used to perform a Confirmatory Factor Analysis.

## 3. Results

### 3.1. Descriptive Statistics

After excluding incomplete questionnaires and ineligible patients (*n* = 12), 229 ACHD patients were retained for final analysis (45% female; response rate: 80.3%). The mean age of ACHD patients was 38.2 ± 12.5 years, and ranged from 18 to 73 years. Demographic characteristics are presented in Table 1**.**

Table 2 shows clinical characteristics of the patient population. The severity of CHD was determined according to the Warnes classification based on Task Force 1 of the 32nd Bethesda conference as simple (*n* = 54, 23.6%), intermediate (*n* = 88, 38.4%) and severe (*n* = 72, 31.4%) [19]. Remaining ACHD who did not fit into the Warnes severity code were classified according to the Munich classification system into simple (*n* = 2, 13.3%) and severe forms (*n* = 13, 87.7%) of CHD. Additionally, CHDs were subclassified into six main groups, consisting of (I) complex congenital heart defects (*n* = 75), (II) post-tricuspid shunts (*n* = 18), (III) left ventricular outflow tract obstruction (*n* = 44), (IV) right ventricular outflow tract obstruction (*n* = 39), (V) pre-tricuspid shunts (*n* = 35) and (VI) miscellaneous CHD (*n* = 18). 3.5% of patients (*n* = 8) presented chronic oxygen deficiency with an oxygen saturation < 92%. The majority of ACHD (*n* = 159, 69.4%) have had operative, palliative or repair surgery at the time of the survey. The functional class (FC) of ACHD was determined according to Perloff’s classification, which classifies ACHD based on their symptomatic restrictions [24]. Accordingly, 218 patients were in FC I/II (95.2%), 9 patients in FC III (3.9%) and 2 in FC IV (0.9%). 

### 3.2. Objective 1: Psychometric Properties of the IIQD

#### 3.2.1. Reliability Analysis

Internal consistency was assessed using Cronbach’s Alpha coefficients. As displayed by the coefficients and dedicated latent constructs in Table 3, the observed alpha-coefficients indicated medium to-high reliability values between 0.79 and 0.93 on all four illness identity dimensions. Thus, all IIQD-dimensions showed acceptable and satisfactory reliability with coefficients for the dimensions’ rejection (α = 0.79) and acceptance (α = 0.88), being slightly lower than alpha-coefficients for engulfment (α = 0.93) and enrichment (α = 0.90). It must be taken into account that the scales for engulfment and enrichment were constructed using a higher number of items (7 and 8 items) than rejection and acceptance, respectively (5 items). Since Cronbach’s alpha depends on the number of items, higher values in engulfment and enrichment may be due to a higher number of items constructing the scale dimensions.

#### 3.2.2. Factorial Validity

Since the IIQD for measuring illness identity in patients with chronic conditions is a new diagnostic measure, factorial structure was validated in addition to reliability testing. Prior to testing the hypotheses, a confirmatory factor analysis (CFA) was performed that analyzed the factorial structure in accordance to the previously postulated dimensions. The hypothesized framework of the original scale posited the four-factor model with engulfment, rejection, acceptance and enrichment. Considering the overall model fit for this factorial structure, the CFA did not demonstrate ideal fit (χ^2^ = 825.15, df = 269, *p* < 0.001). This observation was supported by dedicated fit indices like RMSEA, CFI and TLI (RMSEA = 0.09; CFI = 0.850, TLI = 0.833). Thus, the originally-postulated factorial structure by Oris et al. [11] involving four distinct factors could not be replicated in the current sample. 

While nearly every single relationship between the four factors had statistically significant covariances, this relationship was not the case for the covariance between enrichment and engulfment (*p* = 0.05). Having a closer look at standardized regression weights, most of the estimates for engulfment, acceptance and enrichment had estimates above 0.75. For the dimension of rejection, standardized regression weights were considerably lower than for items of all the other dimensions, and ranged between 0.54 and 0.75. Furthermore, the results indicate a low explained variance in the rejection dimension or *R*^2^ = 0.29 for the third item in the scale, namely “I never talk to others about my illness”. This item may, therefore, not be suitable for the factor, and might potentially measure something different than what was intended for the assigned dimension. It should be mentioned that all items for the three dimensions—engulfment, enrichment and acceptance—were highly correlated with their latent constructs, so that deficits in the four-factor-structure are presumably linked only to the dimension of rejection.

### 3.3. Objective 2: Associations Between Illness Identity and Clinical Parameters 

To identify statistical associations between clinical variables and illness identity states, mean differences between individual groups were analyzed using MANOVA-modeling and post-hoc testing. Significant effects were found for Warnes classification (Wilk’s λ = 0.87, F (8, 406) = 3.48, *p* < 0.01, η^2^_P_ = 0.06) and functional classification (Wilk’s λ = 0.87, F (8, 406) = 3.62, *p* < 0.01, η^2^_P_ = 0.06). No significant interaction effect between Warnes and functional classification could be detected on any of the four illness identity dimensions (Wilk’s λ = 0.91, F (12, 537.37) = 1.51, *p* = 0.11, η^2^_P_ = 0.02).

**Figure 2:**
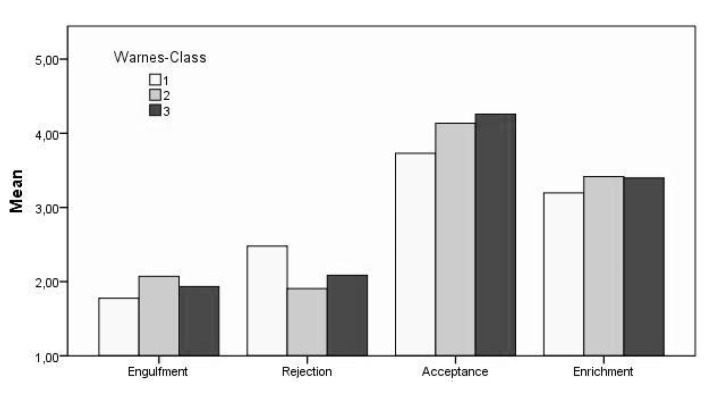
Mean Differences in Illness Identity by Warnes Classification [19].

Looking at between-subjects’ effects in Table 4, the main effects in the Warnes classification do result from significant group differences regarding the dependent variable of acceptance (F (2, 206) = 5.41, *p* < 0.01, η^2^P = 0.05). However, no significant differences could be found for the dimensions of engulfment, rejection and enrichment. Post-hoc analysis using the Tukey-HSD-test revealed significant differences in rejection between the mean scores of Warnes classes 1 (M = 2.47, SD = 0.95) and 2 (M = 1.90, SD = 0.91) on the 1%-level. However, no significant differences were observed between classes 1 and 3 (M = 2.08, SD = 0.91) or 2 and 3 (*p* > 0.05). There were significant differences between the Warnes classes 1 (M = 3.72, SD = 1.01) and 2 (M = 4.13, SD = 0.99), as well as 1 and 3 (M = 4.25, SD = 0.86) regarding their mean scores in acceptance. Thus, there is a tendency for rejection decreasing and acceptance increasing with higher Warnes classes (see Figure 2).

**Figure 3:**
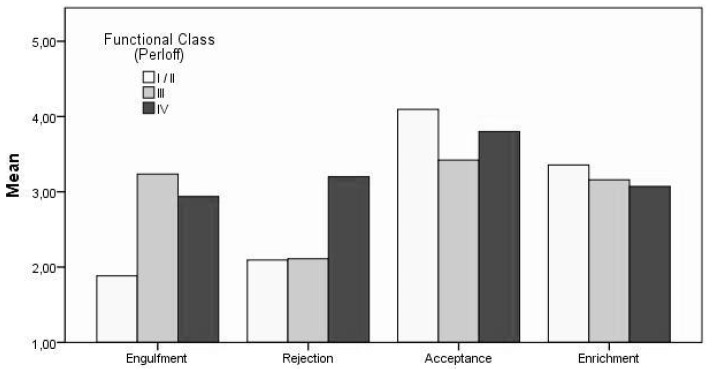
Mean Differences in Illness Identity by Perloff’s Functional Classification [24].

The main effect regarding functional class was found on the dimension of engulfment (F (2, 206) = 10.56, *p* < 0.01, η^2^_P_ = 0.09). Similar to the results of the Warnes classification, no significant differences were observed on the other remaining dimensions (*p* > 0.05). Post-hoc comparisons using the Tukey-HSD-test likewise indicated that the mean engulfment-score for functional class 1 (M = 1.88, SD = 0.97) differed significantly from the mean score of functional class 3 (M = 3.23, SD = 1.19). However, no significant differences were observed for engulfment between functional class groups 1 and 4 (M = 2.93, SD = 1.50) or groups 3 and 4, respectively (*p* < 0.05). Thus, higher functional impairment is positively related to stronger engulfment (see Figure 3). However, higher cardiac severity according to Warnes Classification correlates with increased acceptance and decreased rejection within the present sample.

### 3.4. Objective 3: Associations Between Illness Identity and Emotional Distress

To analyze associations between different dimensions of illness identity and psychological functioning in terms of anxiety and depression, Pearson correlations and multiple regression analysis were calculated. As can be seen in Table 5, several significant correlations between the underlying constructs were obtained. Results indicated significant positive associations between IIQD-dimensions engulfment and rejection in addition to acceptance and enrichment. Moreover, engulfment was negatively correlated with acceptance (*r* (229) = −0.41, *p* < 0.01), as well as enrichment (*r* (229) = −0.13, *p* < 0.05), while rejection had negative associations with acceptance (*r* (229) = −0.49, *p* < 0.01) and enrichment (*r* (229) = −0.27, *p* < 0.01). Considering the HADS-dimensions for psychological functioning, results indicate positive associations between anxiety and engulfment (*r* (229) = 0.61, *p* < 0.01), as well as rejection (r (229) = 0.40, *p* < 0.01). Additionally, anxiety was negatively correlated with acceptance (*r* (229) = −0.39, *p* < 0.01) and enrichment (*r* (229) = −0.27, *p* < 0.01). The same effect directions were obtained for depression, resulting from a strong, positive association between anxiety and depression (*r* (229) = 0.66, *p* < 0.01). To measure the effects of sociodemographic, clinical variables and illness identity on psychological functioning, two models using multiple regression analysis were calculated. 

As can be seen from Table 6, anxiety was significantly predicted by both, clinical classification and illness identity, with heart surgery (*t* = −2.32, *p* = 0.02) and cyanosis (*t* = −2.05, *p* = 0.04) having negative effects. Furthermore, engulfment (*t* = 8.68, *p* < 0.01) and rejection (*t* = 2.52, *p* = 0.01) had positive effects, thus increasing the probability of suffering from anxiety. Different results were obtained for depression as a dependent variable. Here, clinical classification had no significant effects, while age in years had a positive influence of depression (*t* = 2.55, *p* = 0.01). Participants with higher age tended to have significantly more depression than their younger counterparts. Lastly, the illness identity dimensions engulfment (*t* = 9.20, *p* < 0.01) and enrichment (*t* = −4.44, *p* < 0.01) significantly influenced the variability in depression. While engulfment had a positive effect upon depression, enrichment significantly reduced the probability of suffering from depression. 

Within both models, especially dimensions of illness identity successfully explained variance in the dependent variables with the model for depression (*R*^2^ = 0.59), having a slightly better fit than the model for anxiety (*R*^2^ = 0.50).

## 4. Discussion

To the best of our knowledge, this work is the first study to explore the concept of illness identity within a large and representative sample of ACHD at the German Heart Center Munich. Although prior research has addressed the topic of identity within the context of chronic disease management [13,14,25], systematic research on possible antecedents and effects of illness identity in ACHD patients remains insufficient [11]. The purpose of this study was to conceptualize and measure the relationship between illness identity and emotional distress in terms of anxiety and depression. Current results point towards elevated depressive and anxiety symptoms in ACHD. In contrast to previous studies on ACHD, various clinical variables, such as disease complexity, functional status, cyanosis and surgery status, were considered to account for the true severity status of the patients. Findings appeared to be robust when including various confounding factors, such as sociodemographic and clinical parameters. The present study provides evidence that the IIQ/IIQD is a reliable tool to understand and eventually predict the psychological impact of disease to ACHD patients. Illness identity may play a key role in regulating the amount of emotional distress arising in ACHD. Consequently, a patient’s illness identity may potentially predict psychological functioning and ultimately physiological outcomes in ACHD. 

### 4.1. Objective 1: Psychometric Properties of the IIQD

In line with preceding studies, the IIQD proved to be highly reliable with all four scale dimensions indicating Cronbach alpha coefficients above 0.70. While engulfment and rejection were positively interrelated, both dimensions were inversely related to acceptance and enrichment, and vice versa. The associations obtained between the underlying constructs provide evidence for grouping the four illness identity states into functional (acceptance, enrichment) and dysfunctional (engulfment, rejection) ways of illness integration. However, CFA did not demonstrate an ideal model fit in the underlying sample, indicating that four illness identity states, as originally postulated by Oris et al. [11], might be insufficient to fully capture chronic illness integration. Specifically, the rejection dimension might measure heterogeneous constructs indicated by low explained variance, and needs to be further elaborated in future research. 

### 4.2. Objective 2: Associations Between Illness Identity and Clinical Parameters

Mean differences in illness identity were found with respect to disease severity. Against expectations, rather complex heart defects according to Warnes [19] were associated with decreasing tendencies of rejection, as well as higher acceptance rates. Patients with complex heart defects may be more prone to experiencing medical complications throughout life, which forces them to face reality as it is. When additionally looking at the functional and cyanotic status of patients, higher functional limitations and cyanosis correlate positively with engulfment rates. Since these parameters are based on symptomatic restrictions in everyday life, greater functional impairment may affect all important life domains (e.g., family, work, diet, partnership). 

### 4.3. Objective 3: Associations Between Illness Identity and Emotional Distress 

Generally, the present results indicate that the majority of ACHD were able to accept their illness. Since most patients were recruited in the outpatient department, they are probably able to successfully master their everyday lives and engage in their adult responsibilities [11,26]. This situation also explains why most patients had a lower functional status in the current sample. However, patients presented heightened scores of depressive and anxiety symptoms. Hence, present findings confirm the current state of research on the increased prevalence of emotional distress in ACHD [5]. Although patients may function well in day-to-day life, their unrecognized emotional distress may have detrimental effects upon their cardiovascular health. The under-diagnosis of mental health issues and a lack of psychosocial support for ACHD have become increasingly recognized [27]. There are different reasons for this variance. First, clinicians may place their primary focus on the medical treatment of CHD, and might not be aware that patients experience significant psychological problems. On the other side, patients themselves might be unaware of their emotional symptoms, and might not bring their concerns to clinicians’ attention [28]. Further, specialized mental health professionals in the field of CHD are still lacking, since psycho-cardiology in ACHD is a young scientific discipline [29]. 

Clinically-relevant relations between illness identity and emotional functioning were observed consistent with prior expectations. Accordingly, dysfunctional illness identity states were associated with higher emotional distress, while functional illness identity states correlated with better psychological functioning. While no associations could be observed between disease severity and psychological functioning, illness identity accounted for unique differences in psychological functioning, regardless of the underlying disease severity. Hence, disease severity was associated with illness identity, which in turn influenced psychological functioning in ACHD. Closer inspection of the relations between the four illness identity states and their psychological outcomes revealed that engulfment significantly predicted depression and anxiety in ACHD. Patients who are consumed by their illness may probably experience more limitations in daily functions and greater concern in general [11]. Evidence suggests that depression leads to increased threat appraisals [8], which explains the common comorbidity with anxiety [10,28]. 

While health-related anxiety can be beneficial by promoting health awareness and treatment adherence, extreme heart-focused anxiety might further elicit feelings of engulfment and create a vicious cycle of worry and fear [30]. The constellation of depression and anxiety in engulfed patients is especially alarming, because additive effects with a three-fold increased risk of all-cause mortality have been documented elsewhere [9]. Rejection was significantly associated with increased anxiety, but unrelated to depression. Present findings indicate though that adverse effects of rejection on psychological functioning were less evident than adverse effects of engulfment. This form of illness integration might be used by patients who perceive their illness as a threat to their identity, and try to escape the stresses of CHD by suppression, denial or self-distraction [13]. This perception may be initially helpful to temporarily adjust, but long-term effects may prevent patients from applying problem-focused coping strategies [31]. It has been shown that rejection and fear mutually interact: by avoiding confrontation, the perceived threat increases, which in turn leads to greater fear and more rejection [32,33]. Over time, avoidance is known to be associated with continued emotional distress, increased noncompliance to medical regimens, and worse health outcomes [34,35]. In this study, acceptance and enrichment were linked to less depressive symptoms, while the magnitude of effect was higher in enrichment. In line with previous findings, individuals who integrate their illness as an integral part of self might take a more active role in their health management, and have a higher sense of controllability. Controllability is considered a critical component in chronic disease management [36]. It is, therefore, comprehensible that adaptive illness integration is linked to lower depression [11]. In fact, “acceptance” and “meaning making” are listed as key processes within the framework of self-management in chronic illness [15]. It is remarkable that both dimensions of adaptive illness integration are unrelated to anxiety. This lack of relationship indicates that anxiety has a two-fold effect when adapting to a chronic illness: While excessive anxiety, as can be found in maladaptive illness identity states, negatively impacts cardiac outcomes and psychological functioning, healthy levels of anxiety might be a prerequisite for properly integrating the illness and taking appropriate steps in monitoring the illness [37].

Current results should be interpreted with caution due to certain limitations. First, the study was cross-sectional in nature, which does not allow any conclusions about the directionality of effects. It remains to be clarified if illness identity is dynamic in nature or divided into four fixed states ad infinitum. However, illness identity may rather be subject to changes in disease progression and severity, eventual health declines and symptomatic vs. asymptomatic periods [38]. To clarify the role of illness identity within a dynamic process, longitudinal research of the psychological variables applied in this study is needed. Second, all questionnaires used in this study were based on self-report answers. Obtained results were, therefore, susceptible to response bias. This bias was minimized by assuring patients that the survey was strictly pseudonymized, and responses were not shared with their treatment providers. Third, since all patients were aware of being part of a behavioral study, this awareness might have led to biased responses known as the Hawthorne Effect [39]. Fourth, methodological limitations need to be noted. The validation of the IIQD has shown that the four dimensions of illness identity are not sufficient to fully cover a patient’s illness experience. In the future, it may be necessary to adapt the IIQ/IIQD and further differentiate the rejection dimension. Fourth, little is known about the relation of illness identity to other psychologically relevant concepts in chronic disease management, such as psychological coping. Coping in the context of heart disease is typically defined according to the Lazarus and Folkman stress and coping paradigm [40]. Lazarus and Folkman suggest two types of coping: emotion-focused coping involves different ways of emotionally handling the illness in order to reduce emotional distress including wishful thinking, denying, diverting attention or accepting. In contrast, problem-focused strategies directly target the source of stress by activating resources, such as active information seeking and medical compliance. [40] From this perspective, illness identity would rather belong to emotion-focused coping, as it places the focus on intrapsychic processes of illness integration. 

Within this framework, behavioral efforts to handle the illness are regarded as consequences of illness identity, and specific relations between illness identity and practical coping styles still need to be revealed to attain a more nuanced understanding of ACHD and eventually establish psychotherapeutic guidelines. 

## 5. Conclusions

The lack of progress in the area of psychosocial support for ACHD is generally attributed to a lack of general theory and methods in assessing psychological adjustment to CHD. This study provides evidence that the concept of illness identity may potentially provide a helpful tool to understand how patients experience their CHD, how this affects their psychological functioning and how they accordingly respond to treatment. Illness identity might become a particularly important target for intervention in order to assist patients in managing their CHD. Patients with maladaptive illness identity might have a higher risk for experiencing emotional distress, and therefore, require additional support. The next challenge is to translate these findings into interventions and eventually establish psychotherapeutic guidelines in terms of a holistic approach to cardiac care for ACHD. 

## Figures and Tables

**Table 1:** Sociodemographic Variables.

Variables	Value
Age, years	38.2 ± 12.5 (18−7318−73)
Gender (*n* = 229), *n* (%)	
Female	103 (45.0)
Male	126 (55.0)
BMI-values (*n* = 229), *n* (%)	
Underweight	10 (4.4)
Normal weight	107 (46.7)
Pre-obesity	8 (3.4)
Obesity	24 (10.4)
Marital status (*n* = 221), *n* (%)	
Married	94 (42.5)
Divorced	4 (1.8)
Engaged	42 (19.0)
Single	80 (36.2)
Widowed	1 (0.5)
Level of school education (*n* = 217), *n* (%)	
No schooling completed	11 (5.1)
Primary school degree	55 (25.3)
Secondary school degree	60 (27.6)
Vocational / polytechnic degree	28 (12.9)
General University Entrance Qualification	63 (29.0)
Financial standing (*n* = 223), *n* (%)	
Poor	21 (9.4)
Fair	61 (27.4)
Good	141 (63.2)

**Table 2:** Clinical Classification.

Variables	Value
Cyanosis (*n* = 229), *n* (%)	
Acyanotic	221 (96.5)
Cyanotic	8 (3.5)
Functional Class (Perloff) (*n* = 229), n (%)	
I/II	218 (95.2)
III	9 (3.9)
IV	2 (.9)
Severity code of CHD according to Warnes classification (*n* = 214), *n* (%)	
Simple	54 (23.6)
Intermediate	88 (38.4)
Severe	72 (31.4)
Severity code of miscellaneous CHD (*n* = 15), *n* (%)	
Simple	2 (13.3)
Severe	13 (86.7)
Leading diagnosis (*n* = 229), *n* (%)	
Complex congenital heart defects	75 (32.8)
Post-tricuspid shunts	18 (7.9)
Left heart malformation	44 (19.2)
Right heart malformation	39 (17.0)
Pre-tricuspid shunts	35 (15.3)
Other	18 (7.9)
Previous heart surgery (*n* = 229), *n* (%)	
No	70 (30.6)
Yes	159 (69.4)

**Table 3:** Reliability and Number of Items of Latent Scales Indicated by Internal Consistency.

Latent Construct	# Items	Cronbach’s α
*IIQD*		
Rejection	5	0.79
Acceptance	5	0.88
Engulfment	8	0.93
Enrichment	7	0.90
*IIQD: German version of Illness Identity Questionnaire*		

**Table 4:** Results of Multivariate and Between-subject Testing.

Effect	Wilks’ λ	F	Sig.	Partial η^2^
Warnes	0.87	3.48	0.001	0.064
Engulfment	-	2.35	0.098	0.022
Rejection	-	2.83	0.061	0.027
Acceptance	-	5.41	0.005	0.050
Enrichment	-	1.38	0.253	0.013
Functional Class (Perloff)	0.87	3.62	0.000	0.067
Engulfment	-	10.56	0.000	0.093
Rejection	-	1.99	0.139	0.019
Acceptance	-	2.18	0.115	0.021
Enrichment	-	.51	0.601	0.005
Warnes * Functional Class (Perloff)	0.916	1.51	0.114	0.029

Sig. = significance. All findings with a *p*-value < 0.05 are considered significant. * = interaction effect between Warnes and functional classification.

**Table 5:** Means (M), Standard Deviations (SD) and Bivariate Pearson-Correlations.

Variable	M	SD	1.	2.	3.	4.	5.	6.	7.
1. Engulfment	1.94	1.01	1						
2. Rejection	2.10	0.94	0.38 **	1					
3. Acceptance	4.06	0.97	−0.41 **	−0.49 **	1				
4. Enrichment	3.34	1.01	−0.13 *	−0.27 **	0.36 **	1			
5. HADS-Anxiety	7.97	4.06	0.61 **	0.40 **	−0.39 **	−0.27 **	1		
6. HADS-Depression	7.91	4.05	0.68 **	0.32 **	−0.41 **	−0.34 **	0.66 **	1	
7. Need for information	3.08	.72	0.11	−0.09	0.04	0.18 **	0.01	−0.01	1

Note. M and SD represent means and standard deviations, respectively. ** indicates *p* < 0.01. * indicates < 0.05.

**Table 6:** Results of Multiple Regression Analysis Using Anxiety and Depression as Dependent Variables Adjusted.

	DV: Anxiety		DV: Depression	
Predictor Variables	B	SE	B	SE
*Socio-Demographics*				
Age, years	0.00	0.02	0.05 *	0.02 *
Gender	0.69	0.47	−0.76	0.43
Marital Status	0.99	0.55	−0.09	0.51
BMI	0.05	0.06	0.07	0.05
Financial standing	−0.20	0.37	−0.48	0.34
School education	−0.04	0.23	0.16	0.21
Professional education	0.36	0.35	−0.44	0.32
*Clinical Classification*				
Warnes	0.21	0.34	0.23	0.31
Functional class (Perloff)	−0.40	0.45	0.81	0.41
Heart surgery	−1.35 *	0.58 *	−0.22	0.53
Cyanosis	−2.33 *	1.13 *	0.17	1.04
*Illness Identity*				
Engulfment	2.45 **	0.28 **	2.39 **	0.26 **
Rejection	0.69 *	0.27 *	−0.06	0.25
Acceptance	0.01	0.28	−0.16	0.25
Enrichment	−0.44	0.23	−0.97 **	0.21 **
*R^2^*	0.50		0.59	
*R^2^*-adjusted	0.46		0.56	

Note. B and SE represent unstandardized regression coefficients (B) and standard errors (SE) respectively; BMI: Body Mass Index; ** indicates *p* < 0.01. * indicates < 0.05.
